# Dentists’ practice and compliance with current guidelines of infective endocarditis prophylaxis- National survey study

**DOI:** 10.4317/jced.58054

**Published:** 2021-07-01

**Authors:** Ivana Šutej, Matej Par, Dragan Lepur, Kristina Peroš, Hrvoje Pintarić, Ivan Alajbeg, Lovro Vuger

**Affiliations:** 1 Assist. Prof. of Pharmacology, School of Dental Medicine, University in Zagreb, Croatia; 2 Postdoctoral fellow of Endodontics and Restorative Dentistry, School of Dental Medicine, University in Zagreb, Croatia; 3 Assoc. Prof. of Infectious Diseases., School of Dental Medicine, University in Zagreb, Croatia; 4 Prof. of Internal Medicine, School of Dental Medicine, University in Zagreb, Croatia; 5 Prof. of Oral Medicine. School of Dental Medicine, University in Zagreb, Croatia; 6 Dentist at private practice, Zagreb Croatia

## Abstract

**Background:**

The objective of this study was to assess the attitude, practice, and knowledge of Croatian dentists regarding infective endocarditis (IE) prophylaxis.

**Material and Methods:**

A cross-sectional, self-reporting questionnaire survey was conducted with the participation of 348 Croatian dentists. The questionnaire was designed to collect information on participants’ work experience, place of work, their attitudes related to the treatment of IE-risk patients, knowledge and adherence to IE antibiotic prophylaxis guidelines.

**Results:**

Knowledge and adherence to the current guidelines decreased with the higher years of experience. Compliance with the current guidelines varied, mostly because of respondents’ insecurity regarding which guidelines to follow. AHA guidelines have been most frequently the first choice (25% participants). Surprisingly, 23% of dentists didn’t follow any of the official guidelines. The majority of participants (68%) have declared a lack of preparedness or willingness to treat the patients at risk of IE. Dentists with specialty or working at university/hospital have shown a higher level of knowledge and preparedness to treat IE-risk patients.

**Conclusions:**

The lack of knowledge of guidelines and consequent inconsistencies in IE antibiotic prophylaxis in Croatian dental practice indicates the need for urgent improvement.

** Key words:**Antibiotic prophylaxis, dentistry, infective endocarditis.

## Introduction

The use of antibiotic prophylaxis (AP) for certain dental/surgical procedures in patients at risk of infective endocarditis (IE) has been practiced for several decades (1). Association between dental intervention and IE incidence is still drawing the attention of clinicians and scientists because results are so far inconclusive (2-5).

No randomized controlled clinical trial has been performed to resolve the role of AP, and there are no human studies showing that it can prevent IE.

Guidelines on antibiotic prophylaxis for IE risk patients under dental treatment were drafted a half a century ago, and are being regularly updated (6-8). However, despite existing guidelines, fear among dental practitioners of treating high-risk patients is far too common. Divergence of AP recommendations, unavailable patient’s medical documentation, unclear communication with the patient’s cardiologist and the nature of IE as uncommon but devastating disease, makes that fear apprehensible (2,9,10). Insecurity accompanied by guidelines diversity also led to an increase of unjustified/inappropriate antibiotic prescribing (11,12).

Therefore, IE prophylaxis still represents a multifaced problem that dentists are faced with, and there is an obvious need for the improvement of daily practice. That improvement cannot be done without determining the key issues in everyday dental practice and identifying the causes for the most common insecurity.

The aim of this study was to assess dentists’ attitudes, knowledge, practice and adherence to the available guidelines on IE prophylaxis in risk patients undergoing dental treatment.

## Material and Methods

This study was based upon a part of the same subject group dataset of previous publication (13) and adds substantially to each other to warrant publication as separate papers.

All analyzed data were collected using a specially designed questionnaire with structured groups of questions with multiple choice answers. A questionnaire was created and used to identify dental medicine doctors’ attitudes, antibiotic prophylaxis practice/preparedness, knowledge, and adherence to antibiotic prophylaxis guidelines. The first part of the questionnaire gathered general information on occupational records, specialty and practice mode (public or private healthcare). The second part consisted of specific questions that provided data about the dentist’s current practice in managing patients at IE risk. The survey was anonymous. The appropriate choice of antibiotic was set according to the prescription pattern in Croatia, which is amoxicillin and amoxicillin with clavulanic acid (14,15).

All dentists registered in the Croatian Dental Chamber were invited to participate. Participation call was sent by an official e-mail invitation through members’ mailing list. Second calls were sent in a one-week frequency until the month from the first call. The number of registered dentists was obtained from the Croatian Institute of Public Health (CIPH) official annual report.

Statistical procedures used: Descriptive statistics were used to describe the study sample and represent the differences in frequencies of answers between groups. Chi-square test and Z-test with Bonferroni adjustment for multiple comparisons were used to compare proportions among different levels of categorical variables. To evaluate the association between work experience and proper prescription of IE prophylaxis, the participants were divided into groups spanning an age range of five years, and mean values of age for each group was correlated to the corresponding percentage of correct answers using a linear regression model and Pearson’s correlation analysis. Statistical analysis was performed using SPSS 20.0 (SPSS Inc., Chicago, IL, USA). An overall level of statistical significance for all analyses was set at α = 0.05.

The study was approved by the Ethics Committee of the School of Dental medicine, University of Zagreb, Croatia, Europe, Ethical protocol number (approval No. 05-PA-30-IV-2/2019) and was done in accordance with the World Medical Declaration of Helsinki.

## Results

All answers on experience and attitude to treat IE-risk patents in the dental setting are shown in Table 1.

**Table 1:**
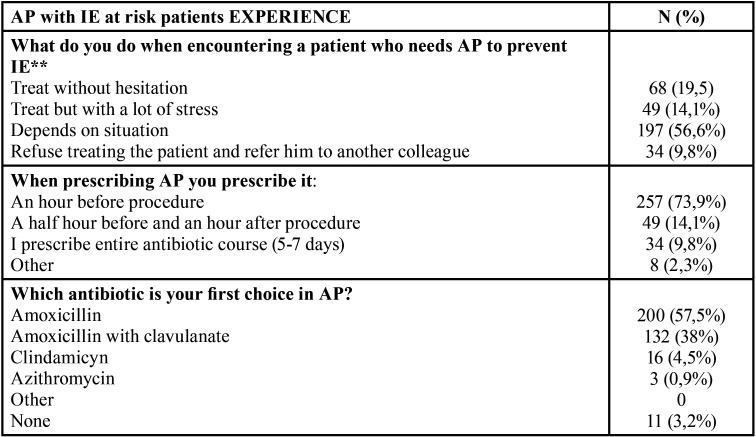
Subjects’ answers on experience and practice with infective endocarditis risk patients.

-Demographic and practice-related characteristics of the respondents

A total of 348 dentists completed the questionnaire. As the total number of dentists registered in the official public records of the Croatian Institute of Public Health at the time of data collection was 2369, an overall national response rate amounted to 14.5%. The demographic data of the participants are summarized in Table 2.

**Table 2:**
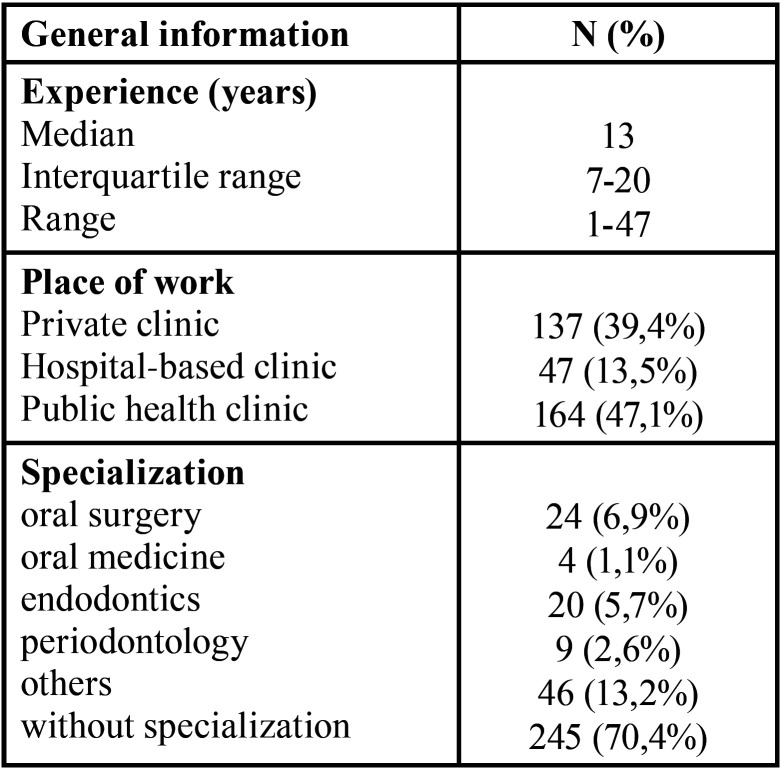
Demographic characteristics of participating dentists.

-Knowledge of IE AP

An appropriate answer on antibiotic choice and first-line prescription at the right schedule (2 g single dose 1 hour before the invasive dental procedure) for IE AP was given in significantly higher frequency by dentists working in a hospital/university setting compared to the dentists working in public or private practice (*p* = 0.007). There was a significant negative linear correlation (R2=0.97, *p*<0.001) between the work experience and an improper prescription of prophylaxis. The main misapplication was the choice of antibiotic and discordance in the regime of antibiotic use (half an hour before and 1 hour after dental treatment). Among the dentists with specialization training, a significantly higher proportion of correct prophylaxis prescription was identified for oral surgeons (*p* = 0.001).

-Adherence to guidelines

Adherence to guidelines for IE AP was significantly heterogeneous among the specializations and types of dental practice. Dentists with a specialization and dentists working in a hospital/university setting were significantly more frequently following the AHA guidelines (*p* < 0.001), while being significantly less adherent to the guidelines of the Croatian Cardiac Society (CCS) or recommendations from a colleague (*p* < 0.001) compared to private practitioners.

-Management of patients at high risk of IE

Dentists with a specialization and dentists working in hospital/university were more prepared to work and had significantly more answers to treat without hesitation the patients at risk of IE compared to the dentists without specialization and other types of dental practices (*p* < 0.001). Private and public health practitioners answered in a significantly higher proportion that they hesitated to work with a patient with a high risk of IE compared to the hospital/university dentists. Among respondents, 9.8% (34) explicitly declared that they are unwilling to treat the patients with a high risk of IE.

## Discussion

A high proportion (68%) of all investigated subjects reported hesitation or was not willing to treat at-risk patients. Results also confirmed that education along with experience is an important factor in the management of IE risk patients. Dentists with completed training as a specialist showed better results in managing high-risk patients. Their answers showed better preparedness, better willingness and greater frequency in answering correctly dosing and regiment questions on IE prophylaxis. Explanations for these results are various, and they need further investigations in order to be confirmed.

Work experience and the right choice of a prophylactic antibiotic regiment, in managing high-risk patients, was found to be statistically significant in this study. Dentists with more than 15 years of practice prescribed more frequently wider spectrum antibiotics and also in a higher percentage adhered to outdated guidelines. This result was in accordance with recent studies (16-20), which reported that younger dentists, who recently graduated or have just finished their specialty training, also had better current knowledge. These results indicate that continuous education is an important factor for maintaining good working practice.

Hesitation to treat, accompanied by referring patients to the specialist, was noticed in 56.6 % subjects, while direct answer not to treat at-risk patients was observed in 9.8 % cases. Less prepared, less willing to treat, and more prone to referring at-risk patients to a specialist were dentists that work in private practice. Since private practices are most vulnerable to legal processing due to patient dissatisfaction, causing loss of resources, private dentists tend to refer IE-risk patients to specialists who are more experienced. Similar results were found in a recent national study among French dentists where those working in a private clinic or hospital had better results in answering a questionnaire versus individuals in private practice (19). Not many studies have assessed physicians’ willingness to treat, which represents important considerations for improvement of the management of IE-risk patients. In comparison with results from the study of RYALAT *et al*. (21), our participants answered not to treat at IE risk patients with a twofold higher frequency than their participants (5%) . An explanation for this outcome can be found in fear caused by antibiotic prophylaxis for IE being a controversial subject in the last decade. To overcome this result, as shown in this study, education and experience are of great importance.

Dentists working in a university/hospital setting, as the results from our study showed, were more secure in making decisions regarding treatment of at-risk patients. Among dental specialists, the greatest frequency of correct answers and preparedness to treat was identified among oral surgeons. This result was partially expected due to their higher frequency in practice with at-risk patients amidst other specialties. In addition, oral surgery is frequently set in a hospital or university setting, where practitioners can consult with other colleagues. The other reason could be continuous education, which is more frequent and common among university/hospital workers.

These results confirm that education, along with experience, is one of the main resources for improving at IE risk patients’ management in dental settings which is in accordance with the latest review study on knowledge and compliance of dentists and dental students for prescribing AP for the prevention of IE (22). A good practical example in improving the system is shown in the study by SAVIDI *et al*. (23) with developing guidelines about AP, giving Iranian general dentists problem-orientated clinical practice guidelines matching their own community population’s needs.

Diversity in answers was greatest regarding guidelines. The wide range of regimens prescribed by dentists confirmed that the standard current prophylactic antibiotic regimen has not been widely accepted. Such diversity has been also found in other studies (18,19,21,24,25). During this research, we encountered many inconsistencies in our country. Many societies dealing with oral and dental health, along with dental and hospital institutions in Croatia, haven’t provided guidelines for IE prophylaxis on their webpages, making insecurity in guidelines choice among dentists a real problem. Croatian endodontic society have recently started with an antibiotic awareness campaign, including their use in prophylaxis, for their results are yet to be seen.

It is important to note that guidance should not be the only factor dictating clinical decisions regarding treating IE risk patients. Each decision should be made on a case-specific basis and be thoroughly discussed with the patient to ensure informed consent (26) especially since a recent clinical study determined the presence of bacteria in the bloodstream following dental care with and without preventive use of antibiotics (27). The problem of patient’s lack of education, which must be improved in order to help overcome this issue, should also be addressed (28,29). Additionally, communication between physicians that treat the same patient is of great importance since it has been proved that recommendations made by cardiologists had an important influence on both dentists and patients (12,30). The European Society of Cardiology (ESC) offers detailed advice regarding antibiotic prophylaxis and IE risk reduction and emphasizes that more so than antibiotic prophylaxis, detailed oral hygiene advice and regular dental examinations are paramount in reducing a patient’s risk of IE. Apart from detailed preventative advice regarding the maintenance of their oral hygiene, at-risk patients should also be explained why is perfect oral hygiene of key importance in preventing IE. Dentists should also explain the risks of invasive dental procedures and soft tissue trauma (2,6,12).

This is the first nationwide attempt, to the best of our knowledge, to evaluate knowledge, practice, and attitude toward IE prophylaxis and management of IE - risk patients in dentistry and the adherence to current guidelines among Croatian dentists.

There are a few limitations of this study. Due to the relatively small study sample, the results may not necessarily be representative in general of all Croatian dentists. Respondent bias may include younger respondents more ready taking an online survey and including more specific specialists interested in the topic.

To conclude, patients at risk of infective endocarditis are present in all aspects of dental care practice with a growing tendency. The lack of the knowledge of guidelines and consequent inconsistent in IE antibiotic prophylaxis in Croatian dental practice requires action. This fact, associated with our findings, indicates the need for continuous education of dentists who should be provided with accurate and easily accessible guidelines on IE prophylaxis.
